# Parents’ Experience With a Mobile Health Intervention to Influence Human Papillomavirus Vaccination Decision Making: Mixed Methods Study

**DOI:** 10.2196/30340

**Published:** 2022-02-21

**Authors:** Elisabeth RB Becker, Ross Shegog, Lara S Savas, Erica L Frost, Sharon P Coan, C Mary Healy, Stanley W Spinner, Sally W Vernon

**Affiliations:** 1 University of Texas Health Science Center at Houston Houston, TX United States; 2 Baylor College of Medicine Houston, TX United States; 3 Texas Children's Pediatrics Houston, TX United States

**Keywords:** human papillomavirus, vaccination, user experience, parent, mHealth, HPV, vaccine, HPV vaccine, parenting, pediatrics, sexual health, cervical cancer, adolescents, mHealth, app, application

## Abstract

**Background:**

Human papillomavirus (HPV)-attributed cancers are preventable, yet HPV vaccination rates severely lag behind other adolescent vaccinations. HPVcancerFree (HPVCF) is a mobile health (mHealth) intervention developed to influence parental HPV vaccination decision making by raising awareness of HPV, reducing HPV vaccination barriers, and enabling HPV vaccination scheduling and reminders through a smartphone app. Evaluating the user experience of mHealth interventions is a vital component in assessing their quality and success but tends to be underreported in mHealth intervention evaluation.

**Objective:**

We aimed to evaluate the user experience of HPVCF, an HPV cancer prevention app designed for a pediatric clinic network, using mixed methods data collected from log files, survey measures, and qualitative feedback.

**Methods:**

Study data were evaluated from parents in a large US pediatric clinic network using HPVCF in the treatment study condition of a group randomized controlled trial. Log data captured HPVCF retention and use. Postintervention rating scales and items assessed HPVCF utility, usefulness, understandability, appeal, credibility, and perceived impact. Overall quality was evaluated using the user version of the Mobile Application Rating Scale (uMars). Open-ended responses assessed parent recommendations for HPVCF enhancement.

**Results:**

The 98 parents were mainly female (n=94, 96%), 41 (5.67) years of age, college educated (n=55, 56%), and White and non-Hispanic (n=55, 56%) and had private health insurance for their children (n=75, 77%). Parents used HPVCF 197 times, with the average visit duration approximating 3.5 minutes. The uMARS app quality score was positively skewed (4.2/5.0). Mean ratings were highest for information (4.46 [SD 0.53]) and lowest for engagement (3.74 [SD 0.69]). In addition, of 95 parents, 45 (47%) rated HPVCF as helpful in HPV vaccination decision making and 16 (17%) attributed HPV vaccine initiation to HPVCF. Parents reported that HPVCF increased their awareness (84/95, 88%), knowledge (84/95, 88%), and HPV vaccination intentions (64/95, 67%). Most of the 98 parents rated the 4 HPVCF components as useful (72-92 [73%-94%]). Parents also agreed that HPVCF is clear (86/95, 91%), accurate (86/95, 91%), and more helpful than other HPV vaccine information they had received (89/95, 94%) and that they would recommend it to others (81/95, 85%). In addition, parents suggested ways to increase awareness and engagement with the app, along with opportunities to enhance the content and functionality.

**Conclusions:**

HPVCF was well received by parents and performed well on indicators of quality, usefulness, utility, credibility, and perceived impact. This study contributes a multimethod and multimeasure evaluation to the growing body of literature focused on assessing the user experience of patient-focused technology-mediated applications for HPV education.

## Introduction

### Background

Human papillomavirus (HPV) is a sexually transmitted infection that causes anogenital cancers and oropharyngeal cancers in men and women [1]. HPV is attributed to 630,000 new cancer cases per year worldwide [2] and 44,000 cases per year in the United States [3]. The majority of HPV-attributed cancers can be prevented with a 2-dose 9-valent HPV vaccine [4]. HPV vaccination is recommended for adolescents 11-12 years old, but rates severely lag behind other adolescent vaccinations, such as Tdap and meningococcal vaccines [5]. The Healthy People 2030 goal for HPV series completion is 80% of youth; however, only 60% of 13-17-year-olds have initiated and 40% have completed the HPV series in the United States [6].

National samples in the United States have found that 28% of parents have refused or decided not to get the HPV vaccine for their child and 8% of parents have delayed or put off getting the vaccine [7]. Refusal is associated with parental perceptions that the HPV vaccine is ineffective and harmful, and delay is associated with the parental need for more information [7]. Frequent reasons for HPV vaccine hesitancy also include perceptions that the vaccination is not necessary, a lack of provider recommendation, and a lack of parental knowledge [8]. Despite this, parent intervention can persuade HPV vaccination initiation, as over 85% of parents with a history of delay have reported initiating HPV vaccination or intending to do so after continued counseling and recommendation [7].

Factors at the individual, provider, and clinic levels have been positively associated with HPV vaccination outcomes. Interventions that address parental psychosocial factors (ie, knowledge, beliefs, and outcome expectations), provider behavior (ie, HPV vaccine recommendation), patient-targeted systems (ie, reminder systems), and provider-targeted systems (ie, assessment and feedback) can positively influence HPV vaccination rates [9]. Multimethod strategies demonstrate the highest rates of maintaining increases in HPV vaccination [10].

The use of parent- and patient-focused apps to promote HPV education and vaccination is on the rise [11-14]. Mobile health (mHealth) is rapidly becoming a dominant mode to deliver health education and health promotion interventions [15]. Evaluating the user experience of mHealth interventions is important in assessing their quality, acceptability to users, and application in real-world clinical settings [16-20] but is often underreported [21-23]. User experience is broadly defined as a person’s perceptions and responses resulting from the use or anticipated use of a product, system, or service [24]. There is a lack of consensus on the best methods, measures, or scales to use when assessing mHealth user experience, although this field is maturing, and a number of scales have been developed that focus exclusively on usability [25,26], quality [27,28], and clinically meaningful risks and benefits [29]. The most frequently evaluated domains in assessments of commercially available mHealth apps include the scientific and clinical basis, functionality, usability, accountability, impact, and popularity [23]. Nouri et al’s [30] systematic review of mHealth evaluation criteria found 7 domains that are commonly used: design, content, usability, functionality, ethical issues, security and privacy, and user-perceived value. In addition, government bodies, such as the UK National Health Service, have developed their own mHealth evaluation standards, which include usability and accessibility to ensure they meet the needs of a diverse set of users, including people with disabilities or those with limited technical knowledge, for their health app marketplace [31]. Of 18 scales developed to evaluate the quality of mHealth apps, the Mobile Application Rating Scale (MARS) is the most frequently applied and is the only scale that has a user version for evaluation by nonhealth professionals [32]. A few studies have evaluated the aspects of user experience for patient-focused technology-mediated HPV interventions, including evaluating the usability of a conversational agent for HPV vaccine counseling of parents and college students using a Wizard of Oz methodology [11,33]; evaluating the feasibility, acceptability, and usability of a cervical cancer and HPV educational virtual agent for Hispanic women [34]; and evaluating the usability of a HPV information website for parents and adolescents [35]. These usability evaluations were done as part of the formative design process and did not include users enrolled in a randomized controlled trial (RCT), using the intervention longitudinally on their own. As HPV vaccination misinformation remains a significant public health problem, there is a need for HPV education apps that are usable, useful, and scalable to motivate parents to vaccinate their adolescent children.

### mHealth Intervention: HPVcancerFree

HPVcancerFree (HPVCF) is an iOS- and Android-compatible smartphone app designed for parents of patients aged 10-17 years who have not initiated HPV vaccination. HPVCF is part of a multilevel intervention aimed at increasing HPV vaccination initiation and completion rates in a large US pediatric clinic network [36]. HPVCF was designed to (1) raise awareness of HPV and its prevention, (2) reduce barriers to HPV vaccination, and (3) enable parents to initiate HPV vaccination scheduling and reminders through their smartphone. Preliminary findings have demonstrated potential of HPVCF in changing parental knowledge and perceptions of HPV vaccination [37].

HPVCF was created using user-centered design principles and Intervention Mapping, an evidence- and theory-based systematic framework for developing behavior change interventions [38]. The design steps included (1) literature review and online synchronous text-based focus groups with parents from the pediatric clinic network to assess HPV attitudes, barriers, beliefs, and needs related to a digital behavior change solution [39]: (2) matrices describing target behaviors, psychosocial determinants of behavior, and change objectives; (3) delineation of theoretical methods and practical applications; (4) prototype build; (5) heuristic evaluation and in-house alpha testing; and (6) iterative user testing to assess app content, function, delivery channel, usability, value, desirability, and adoptability for both design and content.

**Figure 1:**
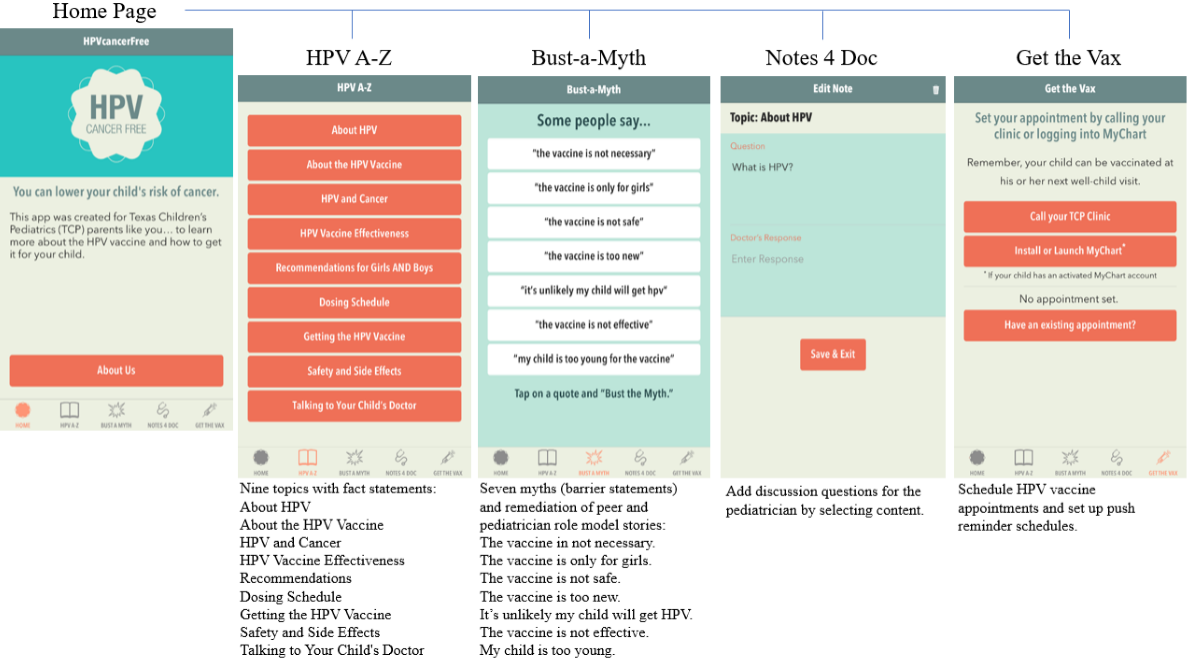
HPVcancerFree (HPVCF) components. HPV: human papillomavirus.

HPVCF contains 4 self-tailored components: (1) HPV A-Z, a compendium of 9 content domains providing facts about HPV and the HPV vaccine; (2) Bust-a-Myth, 7 educational modules, including peer and health care provider testimonials addressing the most salient HPV vaccination barriers; (3) Notes 4 Doc, a medium to facilitate communication with health care providers about the HPV vaccine; and (4) Get the Vax, a feature to schedule HPV vaccination appointments and receive tailored reminders (Figure 1). There were 77 app pages and links that parents had unlimited access to. HPVCF was designed for user-centric navigation and so did not prescribe an intended user path.

The purpose of this study is to evaluate the user experience of HPVCF, an HPV cancer prevention app designed for a pediatric clinic network, using mixed methods data collected from log files, survey measures, and qualitative feedback.

## Methods

### Study Design and Ethics Approval

Study data were drawn from parents who used HPVCF in a group RCT assessing HPVCF effectiveness within a large pediatric clinic network in Texas, USA, and completed a postintervention experience survey [37]. The study occurred between September 2017 and March 2019, where the 51 network clinics were randomized to either the treatment (HPVCF with usual care) or a comparison (usual care only) study condition. Parents in the 26 treatment clinics represented the analytic sample for this user experience evaluation. These parents were given instruction and links to download HPVCF from the Apple App Store (iPhone users) or Google Play Store (Android users). They were given a personal ID to enter the first time they launched HPVCF for tracking purposes. Study protocols were approved by the institutional review board at the University of Texas Health Science Center at Houston (HSC-SPH-15-0202).

### Study Inclusion Criteria and Recruitment

Eligibility for the study included (1) having a 10-to-17-year-old child who was a patient in the clinic network, (2) having a child that had not initiated HPV vaccination, and (3) the ability to speak and write in English. Parents who had an eligible child were invited to participate in the study via patient health record portal invitations, flyers in the clinic waiting rooms, and posts on the clinic network Facebook page. Recruitment for the study took place on a rolling basis from September 2017 to September 2018. Each parent participated in the intervention for 5 months between September 2017 and March 2019, depending on when they were recruited and enrolled. Parent completed a presurvey before they were given access to the intervention and a postsurvey, which included an experience assessment, at the conclusion of their intervention time frame.

### HPVCF Onboarding, Use, and Retention

HPVCF use data were gathered over the course of the 5-month intervention from log files, including total number of visits, number of visits per participant, actions (viewing an app page or link) per visit, and visit duration. A back-end data capture system (Matomo) [40] collected time-stamped use by participant. Actions were triggered when the parent visited a new app page.

#### Experience

HPVCF user experience was assessed with a postintervention survey using a quality rating scale, survey items, and an open-ended response item for recommended enhancements. The survey items included utility, perceived impact, component usefulness, clarity, credibility, and motivational appeal.

#### App Quality

App quality was assessed by the user version of the Mobile Application Rating Scale (uMARS) [28]. uMARS is a reliable mHealth quality measure comprising 3 separate components: an app quality mean score, an app subjective quality scale, and perceived impact items. The app quality mean score contains 16 items evaluating 4 subscales: engagement (5 items), functionality (4 items), aesthetics (3 items), and information (4 items) on a 5-point response from 1 for “inadequate” to 5 for “excellent” and N/A if an app component is not used. uMARS has consistent internal consistency (Cronbach α=.90) for all subscales (engagement α=.80; functionality α=.70; aesthetics α=.71; information α=.78) [28]. The app quality score was calculated by averaging the combined scores for each of the 4 subscales (engagement, functionality, aesthetics, and information).

### Survey Items

#### Utility

Two utility items assessed whether HPVCF information helped parents decide to get the HPV vaccine for their child (no, yes, no opinion) and whether parents got their child the HPV vaccine as a result of using HPVCF (no, yes, no opinion). These items were adapted from prior surveys used with patient-focused digital behavior change interventions in clinic and school settings [41-43].

#### Perceived Impact

Perceived impact was assessed with 5 items on user perceptions of HPVCF. These modified perceived impact uMARS items measured perceptions of change in awareness of HPV and the HPV vaccine, knowledge of HPV and the HPV vaccine, attitudes of HPV and the HPV vaccine, intentions to get their child the HPV vaccine, and communication with the child’s pediatrician about the HPV vaccine. These items were evaluated on a 4-point scale with response options “strongly disagree,” “somewhat disagree,” “somewhat agree,” and “strongly agree” [28]. For analysis, “somewhat agree” and “strongly agree” response options were collapsed into an “agreement” category.

#### Usefulness

Usefulness was assessed using ratings of 4 HPVCF components (HPV A-Z, Bust-a-Myth, Notes 4 Doc, and Get the Vax) with response options “did not use,” “not very useful,” “somewhat useful,” “very useful,” and “do not recall.” For analysis, “very useful” and “somewhat useful” were collapsed into an “agreement” category.

#### Clarity

Clarity was assessed with a single item on whether the goal of HPVCF was clear (no, yes, no opinion).

#### Credibility

Credibility was assessed using 1 rating of accuracy of HPVCF content (inaccurate, accurate, no opinion) and 1 rating of trustworthiness of HPVCF information (cannot be trusted, can be trusted, no opinion).

#### Motivational Appeal

Motivational appeal was assessed using 3 ratings: whether parents would use HPVCF again (no, yes, no opinion), a comparison of the helpfulness of HPVCF content against other HPV content received (less helpful, as helpful, more helpful), and the extent to which parents would recommend HPVCF to others who might benefit from it (few people, several people, many people, everyone). These items were adapted from prior surveys used with patient-focused digital behavior change interventions in clinic and school settings [41-43].

#### Recommended Enhancements

Recommended enhancements were solicited from an open-ended question, “What would make the HPVCF app more appealing so that parents would want to use it?” adapted from prior surveys used with patient-focused digital behavior change interventions in clinic and school settings [41-43].

### Demographics

Parent sociodemographic variables were gathered from preintervention survey items at the start of the 5-month intervention. The parent sociodemographic variables included age, number of adolescent children, sex, race, ethnicity, education, child’s health insurance status, and baseline HPV vaccination intention.

## Results

### HPVCF Onboarding, Use, and Retention

**Figure 2:**
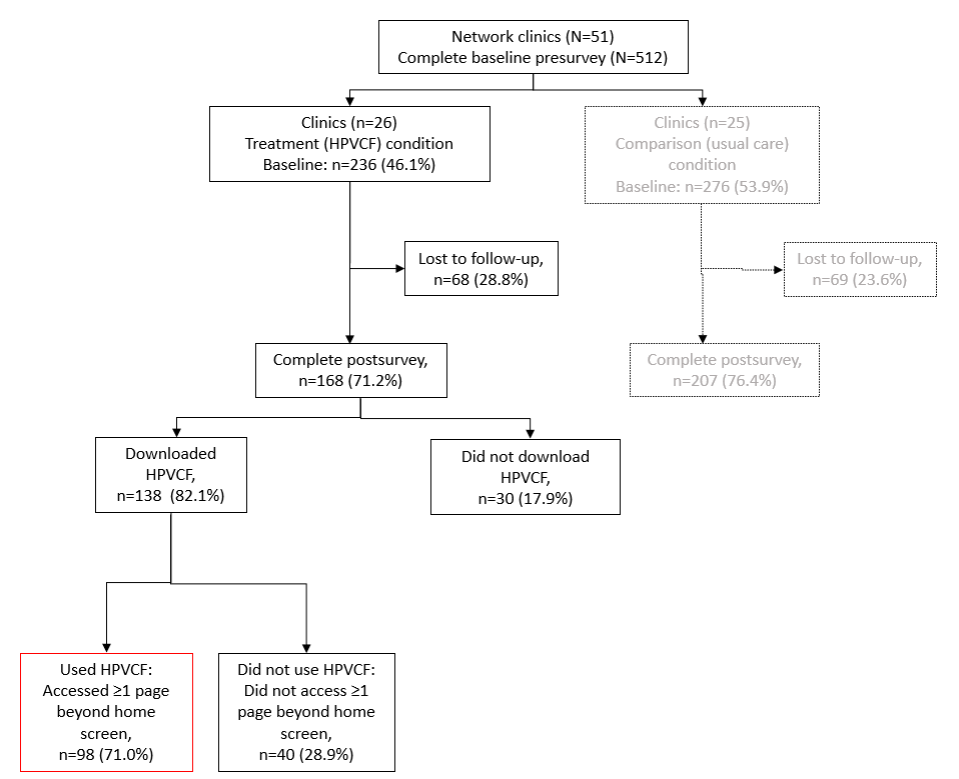
Recruitment and retention. HPV: human papillomavirus; HPVCF: HPVcancerFree.

In total, 168 parents completed the postintervention survey, of whom 98 (58.3%) were included in this experience analysis as they also downloaded and used the intervention (viewed at least 1 page past the home screen on any visit; Figure 2).

Parents had a mean age of 41 years, and the majority were female (94/98, 96%), college graduates (55/98, 56%), and White and non-Hispanic (55/98, 56%) and had private health insurance for their children (75/98, 77%); see Table 1. Most parents had 1 child between 10 and 17 years of age, with a range of 1-4 children in that age group. At baseline, of 98 parents, 12 (12%) reported that they “don’t intend” to vaccinate their child for HPV, 40 (41%) “definitely” planned to, and 46 (46%) were unsure (“haven’t thought of it,” “considering,” and “will probably get”). These demographics reflected the RCT sample where parents had a mean age of 41 years, were majority female (358/375, 95.5%), were college graduates (233/375, 62.1%), and identified as White and non-Hispanic (210/375, 56%). Further, these results approximate the demographic characteristics of the clinic network population where among children 10-17 years old, 45% are White and non-Hispanic and 80% have private health insurance.

Parents visited HPVCF 197 times during the study period (Table 2). Most parents used HPVCF once (45/98, 46%) or twice (28/98, 29%) with a range of 1-8 visits. During a single visit, 2-84 actions occurred with a mode of 3 actions. The average visit duration was 3 minutes and 27 seconds with a mode of 24 seconds and a range from 3 seconds to just under 27 minutes. Of the 4 HPVCF main component pages, HPV A-Z (370 views) was visited most often by parents, followed by Bust-a-Myth (273 views), Get the Vax (173 views), and Notes 4 Doc (110 views).

**Table 1:** Parent demographics (N=98).

Characteristic		Value
**Age (years), mean (SD); range**		41.23 (5.67); 26-54
**Number of adolescent children^a^, mean (SD); range; mode**		1.42 (0.62); 1-4; 1
**Parent sex, n (%)**		
	Male	4 (4)
	Female	94 (96)
**Parent race and ethnicity, n (%)**		
	White, non-Hispanic	55 (56)
	Black or African American, non-Hispanic	7 (7)
	Hispanic	30 (31)
	Asian	4 (4)
	Other	2 (2)
**Parent education, n (%)**		
	Some high school	1 (1)
	High school graduate or General Educational Development (GED)	7 (7)
	Some college	35 (36)
	College graduate	25 (25)
	Graduate or professional degree	30 (31)
**Child/children’s health insurance status^b^, n (%)**		
	Private health insurance	75 (77)
	Medicaid/Medicare/State Children's Health Insurance Program	20 (20)
	Uninsured; no coverage of any type	3 (3)
**Parent baseline HPV^c^ vaccination intention, n (%)**		
	Haven’t thought of it	8 (8)
	Considering	19 (19)
	Will probably get	19 (19)
	Definitely	40 (41)
	Don’t intend	12 (12)

^a^10-17 years old.

^b^Response options are inclusive.

^c^HPV: human papillomavirus.

**Table 2:** HPVcancerFree (HPVCF) use (N=98).

Visit details		Value
**Total number of separate visits^a^ (all parents)**		197
**Number of visits^a^ per parent, mean (SD); range**		2 (1.25); 1-8
**Distribution of parent visits, n (%)**		
	1 visit	45 (46)
	2 visits	28 (29)
	3 visits	13 (13)
	4 visits	10 (10)
	5 visits	2 (2)
	8 visits	1 (1)
**Actions^b^ per visit, mean (SD); range; mode**		11 (10); 2-84; 3
**Visit duration (seconds), mean (SD); range; mode**		207 (249); 3-1601; 24
**Total views by main component page^c^, n**		
	HPV^d^ A-Z	370
	Bust-a-Myth	273
	Notes 4 Doc	110
	Get the Vax	173

^a^A visit was defined as viewing at least 1 page past the home screen.

^b^An action was defined as viewing an app page or link. There were 77 app pages and links in total, which could be viewed unlimited times.

^c^The main component pages could be visited unlimited times while visiting the app.

^d^HPV: human papillomavirus.

#### App Quality

The uMARS app quality rating was 4.2/5.0 (Table 3). The uMARS information subscale had the highest mean rating (4.46 [SD 0.53]), followed by functionality (4.32 [SD 0.65]), aesthetics (4.30 [SD 0.57]), and engagement (3.74 [SD 0.69]).

**Table 3:** User version of the Mobile Application Rating Scale (uMARS) mean scores (N=95).

Subscale^a^	Mean (SD)
Engagement (5 items)	3.74 (0.69)
Functionality (4 items)	4.32 (0.65)
Aesthetics (3 items)	4.30 (0.57)
Information (4 items)	4.46 (0.53)
Overall quality^b^ (from subscales)	4.20 (0.48)

^a^Items in the subscale measured on a 5-point response scale from 1 for “inadequate” to 5 for “excellent” and N/A if an app component was not used.

^b^Calculated by averaging the combined scores for each of the 4 subscales (engagement, functionality, aesthetics, and information).

#### Utility, Perceived Impact, Usefulness, Clarity, Credibility, and Appeal

##### Utility

Overall, 45 (47%) of 95 parents rated HPVCF as helping them decide to get their child the HPV vaccine, and 16 (17%) responded that they got their child the HPV vaccine as a result of HPVCF (Table 4).

**Table 4:** Parent agreement on utility, perceived impact, usefulness, clarity, credibility, and appeal (N=95).

User experience survey parameter		Agreement, n (%)
**Utility^a^**		
	The information I got from HPVCF^b^ helped me decide to get my child the HPV^c^ vaccine.	45 (47)
	I got my child the HPV vaccine as a result of using the HPVCF app.	16 (17)
**Perceived impact of HPVCF^d^**		
	Increased my awareness of HPV and HPV vaccine.	84 (88)
	Increased my knowledge of HPV and HPV vaccine.	84 (88)
	Changed my attitudes of HPV and HPV vaccine.	54 (57)
	Increased my intentions to get my child the HPV vaccine.	64 (67)
	Encouraged me to talk to my child’s pediatrician about the HPV vaccine.	65 (68)
**Usefulness by component^e^**		
	HPV A-Z	92 (94)
	Bust-a-Myth	88 (90)
	Notes 4 Doc	72 (73)
	Get the Vax	73 (75)
**Clarity^c^**		
	The goal/purpose of the HPVCF app was clear.	86 (91)
**Credibility**		
	I think information I got from the HPVCF app was accurate.^f^	86 (91)
	I think the information I got from the HPVCF app can be trusted.^g^	85 (90)
**Appeal**		
	I would use HPVCF again.^c^	63 (66)
	Compared to other information I have seen about the HPV the HPVCF app is as or more helpful.^h^	89 (94)
	I would recommend HPVCF to others.^d^	81 (85)

^a^Responded “yes” as opposed to “no” or “no opinion.”

^b^HPVCF: HPVcancerFree.

^c^HPV: human papillomavirus.

^d^Includes “somewhat agree” and “strongly agree” response options.

^e^N=98; combined responses of “very useful” and “somewhat useful.” Combined percentage responses of “did not use” and “do not recall” were as follows: HPV A-Z (5/98, 5%), Bust-a-Myth (5/98, 5%), Notes 4 Doc (19/98, 19%), and Get the Vax (20/98, 20%).

^f^Rated as “accurate” as opposed to “inaccurate” or “no opinion.”

^g^Rated as “can be trusted” as opposed to “cannot be trusted” or “no opinion.”

^h^Rated as “as helpful” or “more helpful” as opposed to “less helpful.”

##### Perceived Impact

Most parents (64/95, 67%) agreed that HPVCF increased their intentions to get their child the HPV vaccine. Parents reported that HPVCF positively impacted their awareness (84/95, 88%), knowledge (84/95, 88%), and attitudes (54/95, 57%) about HPV and the HPV vaccine and encouraged them to discuss the HPV vaccine with their child’s pediatrician (65/95, 68%); see Table 4.

##### Component Usefulness

Most parents rated the 4 HPVCF components as useful (Table 4). HPV A-Z (92/98, 94%) scored the highest, followed by Bust-a-Myth (88/98, 90%), Get the Vax (73/98, 75%), and Notes 4 Doc (72/98, 73%). Most parents used Bust-a-Myth and HPV A-Z (both ≥95%); however, 20 (20%) and 19 (19%) of 98 parents reported not using the Get the Vax and Notes 4 Doc components, respectively.

##### Clarity, Credibility, and Appeal

The majority of parents rated the purpose of HPVCF as clear (86/95, 91%) and that the information in HPVCF was accurate (86/95, 91%) and can be trusted (85/95, 89%). Parents also agreed that they would use HPVCF again (63/95, 66%), that it was more helpful than other information they had seen about HPV and the HPV vaccine (89/95, 94%), and that they would recommend HPVCF to others (81/95, 85%); see Table 4.

### Recommended Enhancements

Qualitative feedback gathered on how to improve HPVCF included themes of increasing awareness and engagement with the app and enhancing the content and functionality.

#### Increasing Awareness and Engagement

Parents commented that they forgot to use the app after the initial download and needed a reminder from the app, pediatrician, or clinic to use HPVCF:

It's been a long time since I used the app, so I don't remember if there was a function to remind the user in the future to make an appointment etc. That would prompt the user to reopen the app. I read all the information the first time I opened it and didn't open it again, so I've forgotten much of it now.

Due to a lack of repeated engagement, some parents reported they had forgotten much of the information they had originally reviewed. Parents suggested improving engagement by having push notifications with HPV facts instead of having to open and use the app to obtain information. Further, to improve marketability, one parent recommended incorporating HPVCF content into a broader app that included topics outside of HPV.

Parents also suggested making HPVCF more interactive and entertaining, especially by providing opportunities to engage and speak with adolescents about HPV and sexual health. To make HPVCF more adolescent friendly, parents suggested adding animations and games.

#### Enhancing Content and Functionality

To improve the content, parents suggested offering parent testimonials highlighting their struggle to decide to vaccinate and how they used HPVCF to make an informed decision:

I think offering parent testimonials about their struggle to decide to vaccinate and how they used the info offered to help make an educated decision.

Some parents were dissatisfied with the presentation of HPV’s long-term effects, expecting to see more in-depth information and studies about complications and side effects of the vaccine:

This app, as a parent, did not give me the type of information I would want and need about HPV. I would prefer more information on the age of the vaccine. Credible studies completed. New research and side effects. More of that information would help. If the vaccine is less than 10 years old, I want more information on studies.

Parents wanted a tailored reminder system that conveyed information regarding their child’s HPV vaccination status and recommendation:

[I would suggest] some type of electronic reminder from the doctor to review the app. I downloaded the app for the survey, reviewed it, but then forgot about it. Maybe the doctor's office can send email to parents of 9-, 10-, and 11-year-old patients. Also, [a] reminder should have information on [the] child's status (ie, for informational purposes only; needs first dose; received first dose, time for second; etc).

Parents noted some issues with the available functionality, such as the app not storing their appointment information caused by usability issues with the design. For future iterations, parents suggested the ability to share information in the app with existing social media outlets:

[It would be helpful if the app would] allow for flagging/sharing individual items to foster organic awareness through existing social outlets?

## Discussion

### Principal Findings, Strengths, and Limitations

This study evaluated the user experience of HPVCF, an HPV cancer prevention app designed for parents with children belonging to a large urban pediatric clinic network in the United States. Parents viewed HPVCF as having high quality, utility, and perceived impact. HPVCF quality ratings were robust (4.2) compared to quality scores of 2.4-4.6 for mHealth apps focused on prostate cancer risk [44], Alzheimer disease [45], alcohol use [46], occupational therapy [47], orthopedic rehabilitation [48], and medication adherence [49]. HPVCF quality was rated the highest for information and lowest for engagement subscales. This is consistent with the parents’ perceptions of HPVCF information as accurate and trustworthy, while also acknowledging the need to enhance functions and features. The positively skewed quality rating is consistent with a considerable number of parents (16/95, 17%) attributing their child’s HPV vaccination to HPVCF and the majority of parents (64/95, 67%) attributing HPVCF to increasing their intentions to vaccinate their child. This is promising, considering that the period of participation in the study was only 5 months, which is inadequate to fully track vaccinations through annual well visits. The study design was insufficient to determine whether these perceptions and vaccinations were significantly different from trends in parents not exposed to HPVCF, but it does appear that HPVCF was sufficiently persuasive to move at least a sample of parents to action. It is also unclear whether, in these instances, HPVCF was directly associated with vaccination or mediated through greater pediatrician dialogue that promoted vaccination.

Interestingly, 63 (66%) of 95 parents reported that they would use the app again, but log data indicated that 45 (46%) of 98 parents only used the app once. Triangulating these findings with information obtained in the qualitative feedback suggests possible reasons for this, including forgetting about the app after initial download, only using HPVCF on an as-needed basis, or no longer needing the app since it fulfilled its intended use after a single visit. Future iterations could be strengthened to help parents reengage by utilizing pediatricians and clinic staff to incorporate reminders as part of their standard communication. Exploring adjunct functionality that offers HPVCF information in more compact ways (ie, push notifications, text messages) may prove beneficial as most parents only looked at a few pages or links for a brief time (under 3.5 minutes).

Parents perceived HPVCF as more impactful for increasing awareness and knowledge than in changing attitudes about HPV. Knowledge is necessary but not necessarily sufficient to elicit behavior change. Negative attitudes around HPV vaccine safety are particularly pervasive [9], and strategies that are personalized, tailored, and require engagement beyond passive education may be needed to modify attitudes [50]. Concerns about HPV vaccination may have extended to HPVCF itself, with some parents feeling that HPVCF is biased in its portrayal of the long-term risks and safety of the HPV vaccine, despite high credibility ratings. A further behavioral impact was that the majority of parents also reported that HPVCF prompts greater communication with their pediatrician. This is an important adjunct function that helps the parent engage more competently with the pediatrician and provides the pediatrician with an opportunity to educate the parent during “teachable moments” at the clinic visit.

Importantly, parent engagement with the intervention was low, with about 70 (41.7%) of 168 parents choosing not to use HPVCF, making it difficult to generalize results to the clinic network. The low engagement is partially a reflection of the real-world nature of the study and accompanying challenges of competing for attention in an open market. Future iterations might adopt a more assertive approach by having parents download HPVCF during clinic visits and having clinic staff be more involved in providing reminders for its use. Future studies can explore promotional strategies to motivate parents to use HPVCF.

Additional limitations of this study should be considered. The intervention timeline did not include the back-to-school vaccination period (generally June-September) for many adolescent children, as the intervention took place on a 5-month rolling basis over the course of 1.5 years. This may have affected the parents’ decision to get their adolescents vaccinated. The 5-month intervention timeline also meant that some parents answered postintervention questions weeks after using HPVCF, possibly affecting their ability to accurately recall and report on some survey measures. A further limitation of the study was that the English-only content and the smartphone-based application may have excluded participation from parents with lower socioeconomic status or those who do not speak English. Although smartphone ownership among Americans is high (85% White, 85% Hispanic, 83% Black), there are disparities between Americans who are college educated (93%) and those with a high school diploma or less (75%) [51], and health app usage among low-income, racial minority, and ethnic minority patients in Texas remains low [52]. Future research addressing non-English-speaking and populations with a lower socioeconomic status is recommended. Finally, this analysis did not examine user experience by participant characteristics. However, subsequent analyses explored content-specific patterns of use that underlie psychosocial characteristics of parents [53].

As digital technologies continue to evolve, they stand to provide a paradigmatic shift in how health education and health behavior research are conducted [54], but doing so will require them to be perceived by users as being usable and useful applications. This study contributes a multimethod and multimeasure evaluation to the growing body of literature focused on assessing the user experience of patient-focused technology-mediated applications for HPV education [11,33-35].

### Conclusion

HPVCF was well received by parents and performed well on indicators of quality, usefulness, utility, credibility, and perceived impact. HPVCF contributes to a multimethod and multimeasure evaluation strategy for user experience, which remains underreported in mHealth apps.

## Abbreviations

HPV: human papillomavirus
HPVCF: HPVcancerFree
mHealth: mobile health
RCT: randomized controlled trial
uMARS: user version of the Mobile Application Rating Scale
